# Sphingolipids in the Heart: From Cradle to Grave

**DOI:** 10.3389/fendo.2020.00652

**Published:** 2020-09-15

**Authors:** Anna Kovilakath, Maryam Jamil, Lauren Ashley Cowart

**Affiliations:** ^1^Department of Human and Molecular Genetics, Virginia Commonwealth University, Richmond, VA, United States; ^2^Department of Biochemistry and Molecular Biology and the Massey Cancer Center, Virginia Commonwealth University, Richmond, VA, United States; ^3^Hunter Holmes McGuire Veteran's Affairs Medical Center, Richmond, VA, United States

**Keywords:** sphingolipids, ceramide, sphingosine-1-phosphate, heart development, cardiovascular disease

## Abstract

Cardiovascular diseases are the leading cause of mortality worldwide and this has largely been driven by the increase in metabolic disease in recent decades. Metabolic disease alters metabolism, distribution, and profiles of sphingolipids in multiple organs and tissues; as such, sphingolipid metabolism and signaling have been vigorously studied as contributors to metabolic pathophysiology in various pathological outcomes of obesity, including cardiovascular disease. Much experimental evidence suggests that targeting sphingolipid metabolism may be advantageous in the context of cardiometabolic disease. The heart, however, is a structurally and functionally complex organ where bioactive sphingolipids have been shown not only to mediate pathological processes, but also to contribute to essential functions in cardiogenesis and cardiac function. Additionally, some sphingolipids are protective in the context of ischemia/reperfusion injury. In addition to mechanistic contributions, untargeted lipidomics approaches used in recent years have identified some specific circulating sphingolipids as novel biomarkers in the context of cardiovascular disease. In this review, we summarize recent literature on both deleterious and beneficial contributions of sphingolipids to cardiogenesis and myocardial function as well as recent identification of novel sphingolipid biomarkers for cardiovascular disease risk prediction and diagnosis.

## Introduction

Sphingolipids, which constitute a large and diverse lipid class, were originally recognized over a century ago as structural components of cell membranes. More recently they are recognized as crucial bioactive lipids that regulate many cell processes (1). Sphingolipid biosynthesis commences with condensation of an amino acid with acyl-CoA to yield an amino alcohol, or sphingoid base, which is the defining structural component of the sphingolipid class. The sphingoid base can subsequently be modified by acylation, phosphorylation, glycosylation, and/or addition of multiple headgroups or other functional groups (2, 3). These structural modifications generate hundreds of sphingolipid subspecies involved in most if not all major aspects of cell regulation including cell division and senescence, migration, differentiation, apoptosis, autophagy, nutrient uptake, metabolism, and protein synthesis (1). Commensurate with their multiple regulatory roles, disruption of sphingolipid metabolism has emerged as a component of many diseases including cardiometabolic disease. As such, sphingolipid metabolism may be a suitable therapeutic target in the context of cardiovascular disease (CVD). However, perhaps less appreciated are the constitutive and protective roles of sphingolipids in some contexts including heart development and ischemic injury, and these desirable and homeostatic roles should be considered for both *in vivo* experimental design and, more importantly, developing pharmacologic strategies for clinical use. A comprehensive awareness of both deleterious and beneficial roles of sphingolipids will inform successful therapeutic approaches based on targeting sphingolipid metabolism.

## Sphingolipid Biosynthesis: a Brief Overview

*De novo* synthesis of sphingolipids starts in the endoplasmic reticulum (ER) where the enzyme serine palmitoyltransferase (SPT) catalyzes the condensation of an amino acid with acyl-CoA into 3-ketodihydrophingosine (KDHSph). The second step occurs through 3-Ketodihydrosphingosine reductase, which rapidly converts KDHSph to dihydrosphingosine (DHS). DHS is the first easily detected sphingolipid metabolite and serves as the sphingoid base for synthesis of ceramides and downstream complex sphingolipids. DHS can be phosphorylated (forming DHS-1-phosphate) but more often undergoes N-acylation. This is accomplished by a family of (dihydro)ceramide synthase enzymes (CerS) consisting of 6 isoforms with various enzymological distinctions including partially distinct substrate preferences for the incorporation of fatty acids with different chain lengths (4–6). In mammals the length of the ceramide acyl chain length ranges from medium (12-14C), long (16-20C), very long (22-26C), and ultra-long chain fatty acids (>26C). Dihydroceramide (DHC) is converted into ceramide by DHC desaturase (DES), which introduces a double bond into the sphingoid base. Once formed, ceramide can be hydrolyzed by ceramidase enzymes, yielding sphingosine, which can be reincorporated into ceramides by CerS or phosphorylated by sphingosine kinases (SphK1 and SphK2) to produce sphingosine-1-phosphate. Ceramide can also undergo phosphorylation, yielding ceramide-1-phosphate, or O-acylation, yielding a structure similar to a triacylglycerol (TAG), and similarly, is stored in lipid droplets (7). Most ceramide, however, is shuttled to the Golgi apparatus via vesicular transport or ceramide transport protein (CERT) for further metabolism to complex sphingolipids including glycosphingolipids (GSLs) and sphingomyelins (SM) through the addition of sugars or phosphocholine, respectively. These complex sphingolipids can be catabolized to yield ceramide, which plays an essential role in regulating cell ceramide profiles. A less well-studied pathway of sphingomyelin catabolism generates sphingosylphosphorylcholine (SPC). SPC is composed of a long-chain sphingosine and phosphorylcholine and is essentially lyso-sphingomyelin, thus sharing similar structure with S1P and other lysophospholipids. Phosphorylated sphingoid bases are the only known sphingolipids that can exit the cell sphingolipid pool. This occurs via S1P lyase (SPL) which catabolizes S1P into non-sphingolipid components; fatty aldehyde and ethanolamine phosphate. Because SPL is the only exit from the sphingolipid metabolic pathway, it has been proposed as a major regulator of total cell sphingolipid levels (8).

The diversity of sphingolipid species arises not only from the length of the N-acyl chain of ceramide and its derivatives or the functional groups added to the sphingoid base, but also from the length of the sphingoid base. Synthesis of the sphingoid base occurs through SPT, a multimeric enzyme comprised of catalytic subunits and various regulatory proteins. SPTLC1 and SPTLC2 form the canonical catalytic complex, but SPTLC3 can also be included and/or substitute for SPTLC2. The composition of the SPT complex determines substrate and product specificity. The SPTLC1/SPTLC2 complex condenses serine with palmitoyl-CoA giving rise to canonical sphingolipids with an 18-carbon sphingoid backbone (d18:0 DHS) (9). In contrast, inclusion of SPTLC3 in the SPT complex renders a more promiscuous enzyme, using 14-carbon myristoyl-CoA, 18-carbon stearoyl-CoA, and potentially others. This seems to be regulated at least in part by inclusion of small SPT subunits (ssSPTa and b) in the SPT complex (10). These bases are also incorporated into downstream sphingolipids, though not evenly across all sphingolipid species (11). Importantly, variations in both sphingoid base and N-acyl chain determine biological functions (3, 12). Several proteins, namely, neurite outgrowth inhibitor (Nogo-A/B) and orosomucoid-like proteins (ORMDL) negatively regulate SPT. Upon their respective ablation or inhibition, the resulting heart phenotype drastically varies, suggesting sphingolipid levels from *de novo* synthesis must be within a narrow range to maintain normal heart homeostasis (13–15). In the cardiovascular system, many sphingolipid subclasses and even specific molecules have distinct functions, some of which are desirable and others deleterious; for example, increased ceramide and SM oftentimes with concomitant decrease of S1P have been implicated in dilated cardiomyopathy, diabetic cardiomyopathy (DbCM), ischemic heart disease (IHD), and myocarditis (16–20). Therefore, the alterations that occur in sphingolipid content and profiles in disease contexts have emerged as a central focus in cardiovascular biology.

## Sphingolipids in Heart Development and the Cardiac Conduction System

**Figure 1:**
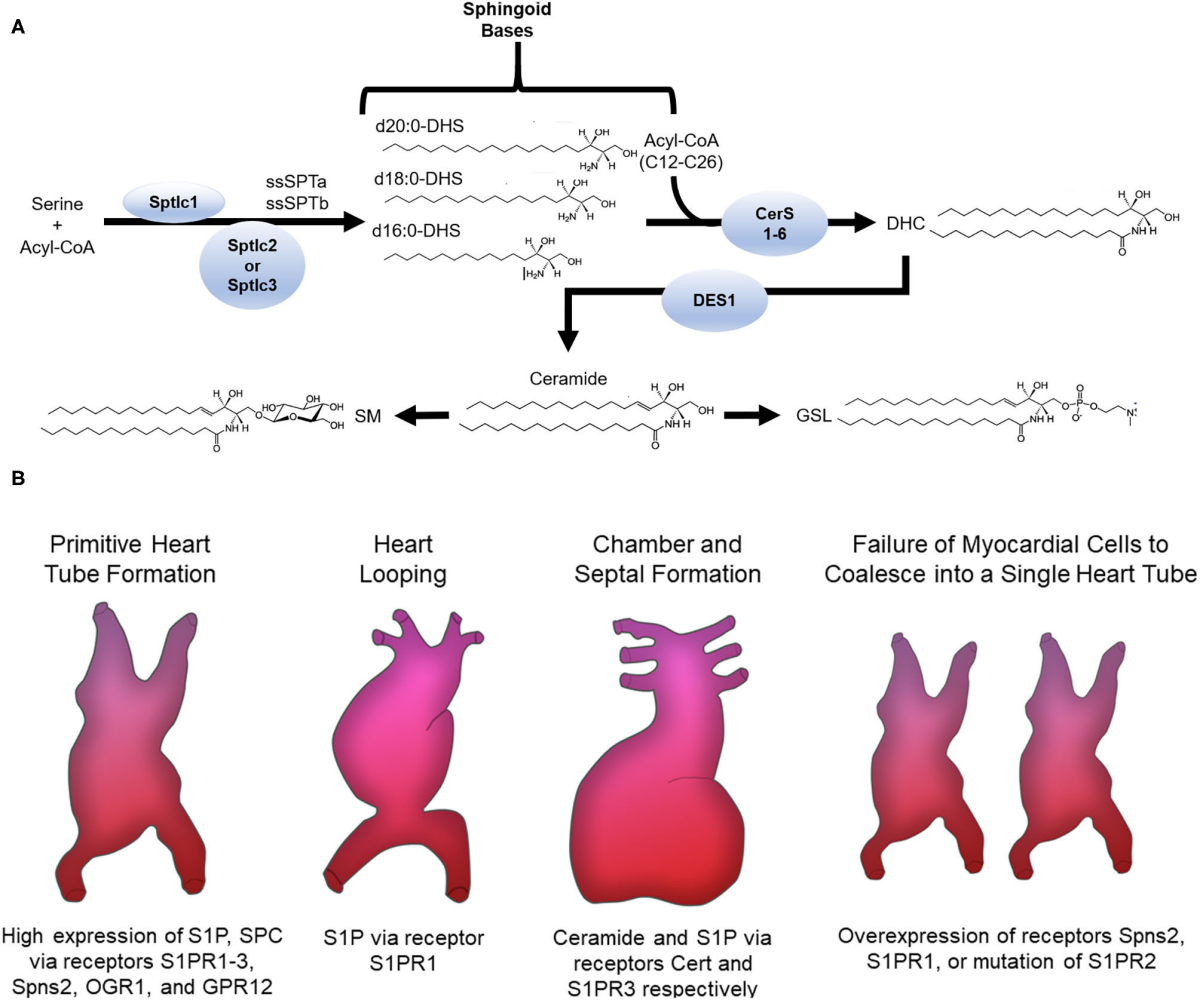
**(A)** Sphingolipid *de novo* synthesis. Overview of sphingolipid structure and metabolism. ssSPTa, small SPT subunit a; ssSPTb, small SPT subunit b; DHS, dihydrosphingosine; CerS, ceramide synthase; DHC, dihydroceramide; DES, DHC desaturase; SM, sphingomyelin; GSL, glycosphingolipid. **(B)** Sphingolipids involved in cardiogenesis. The receptors S1PR1-3, Spns2, OGR1, GPR12 and the sphingolipids S1P and SPC are highly expressed in stem cells that migrate to form the primitive heart tube. Normal expression of S1P via the S1PR1 receptor is needed for normal heart looping. Ceramide and S1P via the Cert and S1PR3 receptors, respectively, are required for chamber and septal formation. Cardia bifida is observed after failure of the myocardial cells to coalesce into one single primitive heart tube. Overexpression of Spns2 or S1PR1 or mutation of S1PR2 causes cardia bifida.

Multiple processes are involved in forming the heart, which is the first functional organ in vertebrate embryos. Cardiogenesis begins with formation and positioning of the primitive heart tube followed by heart looping, and finally chamber and septal formation (Summarized in Figure 1). In the context of cardiovascular biology, sphingolipids, especially ceramides, are most often considered deleterious; however, data show that the sphingosine kinase/sphingosine-1-phosphate signaling pathway is essential for heart development.

Studies in both zebrafish and mice support a regulatory function for sphingosine-1-phosphate (S1P) in formation and positioning of the primitive heart tube. Sphingosine produced by hydrolytic de-acylation of ceramides can be phosphorylated by sphingosine kinases (SphKs) to produce S1P, which signals through endocrine, paracrine, and autocrine mechanisms depending on context (21). For endocrine and paracrine functions S1P is transported into the extracellular milieu through plasma membrane ATP binding cassette family members (ABC) or spinster two (Spns2) transporters where it then signals through one of five different G protein-coupled receptors (S1PR1-5) (21). In Zebrafish, mutations in *s1pr2*, but not any of the other S1P receptors, led to cardia bifida or formation of two laterally positioned hearts (22) (Summarized in Figure 1). This phenotype was also observed in multiple studies with a *spns2* mutant or overexpression of *s1pr1* (22–29). In mice, conditional knockout (KO) of S1PR1 caused embryonic lethality due to ventricular non-compaction, ventricular septal defects, absence of normal increase in the number of cardiomyocytes and decreased myofibril organization (28). These studies suggest S1P signaling components S1PR1, S1PR2, and Spns2 are necessary for cardiomyocyte expansion and myocardial precursor migration to the ventral midline of the embryo where they develop into the primitive heart tube. At later developmental stages, knockdown of the zebrafish gene homolog to *s1pr1* caused an improperly looped heart (30, 31). Another study showed that an *s1pr1* morpholino in zebrafish affected heart valve orientation, an indicator of incorrect looping (28). During the looping process, precursors of cardiac valves—endocardial cushioning (EC) and atrioventricular canal (AV)—are also formed. In mice, S1PR1^−/−^, but not S1PR2 or S1PR3 KO, resulted in embryonic lethality due to severe heart hemorrhaging (32, 33). This may arise from the well-known role of this receptor in maintaining cell-cell contacts in the vascular endothelium (34). In mouse whole embryo cultures, inhibition of sphingosine kinase led to cell death, but elevating S1P levels prevented differentiation of cells into distinct cardiac cell types (35). These studies show that appropriate concentrations of S1P must be maintained for normal development of cardiac valve precursors. It should be noted, however, that affinities of S1P receptors for S1P are in the nanomolar range (e.g., 50–250 nM) and therefore, effects observed with higher concentrations *in vitro* may arise from non-specific activity, for example cross-reactivity with other lysophospholipid receptors, or even gross membrane perturbations due to the detergent properties of S1P. Therefore, care must be taken in interpreting data from experiments using supraphysiological S1P concentrations.

In addition to development, cardiac function also suffers from perturbed sphingolipid synthesis. For example, mouse embryos treated with exogenous S1P exhibited sinus bradycardia (decreased heart rate) (35, 36). In addition, S1PR3 KO mice treated with FTY720, an S1P receptor agonist known to cause sinus bradycardia in humans, did not show altered heart rates, whereas arrhythmias were observed in S1PR3 knock-in (KI) mice (36). S1PR3 is expressed on neural crest-derived atrioventricular nodes (AVN), His bundles, cardiac Purkinje fibers and vascular smooth muscles of the coronary arteries in mice. Extensive studies have suggested S1P plays a multifaceted role as a primary and secondary messenger in regulating both calcium and potassium ion channels (37, 38). This suggests S1P binds its receptor, S1PR3 within the AVN conduction block to regulate intracellular calcium and potassium levels which in turn alter the heart rate (39–41). Taken together these studies show broad roles for S1P receptors in the cardiac conduction system.

SPC displays cross-reactivity to S1P receptors due to its structural similarity to S1P. In addition, SPC also signals via OGR1 and GPR12 receptors (42). SPC, like S1P, is important for heart development, playing a pivotal role for end stage differentiation of committed multipotent cardiovascular progenitors to cardiomyocytes, vascular smooth muscle and endothelial cells (43). Importantly, SPC also induced differentiation of resident cardiac stem cells to cardiomyocytes, a finding which may hold tremendous therapeutic potential (44), as there is currently great interest in therapeutic strategies that leverage the potential for stem cell differentiation to cardiomyocytes in treating cardiac injury. In sum, several lines of evidence point to essential functions of S1P, SPC and their respective receptors in normal heart development and function (summarized in Table 1). Therefore, while sphingolipids have largely been implicated in cardiac pathology, they make essential contributions to cardiogenesis and therefore a broader cognizance of sphingolipids in the heart may benefit efforts to develop sphingolipid-based therapeutic approaches.

**Table 1:** Sphingolipid knockout models and their cardiac tissue phenotypes.

**Animal model**	**Cardiac tissue phenotype**	**References**
Constitutive heterozygous *Sptlc1* knockout & glycosylphosphatidylinositol (GPI)-anchored human lipoprotein lipase transgenic	- Decreased cardiac ceramides comparable to WT mice - Prevention of lipotoxic cardiomyopathy induced by glycosylphosphatidylinositol (GPI)-anchored human lipoprotein lipase knockout	(16)
Cardiomyocyte-specific *Sptlc2* knockout	- Decreased C18:0 and very long chain ceramides - Increased ER stress markers - Increased apoptosis - Upregulation of heart failure markers - Decreased fractional shortening - Thinner cardiac walls	(45, 46)
Constitutive *α-galactosidase A* knockout (Fabry disease)	- Progressive accumulation of globotrioasylceramide in aged mice - Decreased glucosylceramides - No alteration in total ceramide	(47, 48)
Constitutive *Smpd1* knockout	- Accumulation of aSMase in aged mice	(49)
Heterozygous smooth muscle-specific deletion of *Asah1* (acid ceramidase)	- Severe arterial medial calcification in aorta and coronary arteries	(50)
Constitutive heterozygous *SPL* knockout	- Smaller infarct size after ischemic/reperfusion (I/R) injury - Increased S1P - Increased functional recovery after I/R	(51)
Constitutive mutant S*pns2* allele (zebrafish)	- Cardia bifida - Shortened anterior–posterior distance in the ventral pharyngeal arch - Embryonic lethality	(30)
Constitutive *SphK1* knockout	- Decreased S1P levels - Poor animal resuscitation after cardiac arrest - Impaired survival post-resuscitation after cardiac arrest - Increased infarct sizes after I/R	(52, 53)
Constitutive maternal and zygotic *SphK2* knockout (zebrafish)	- Cardia bifida - Failure of cardiac progenitor migration to form primitive heart tube - Decreased S1P levels	(54)
Cardiomyocyte-specific *S1pr1* knockout	- Ventricular non-compaction - Ventricular septal defects - Perinatal lethality - Decreased cardiomyocyte proliferation - Decreased myofibril organization - No alteration in coronary I/R injury	(55)
Cardiomyocyte, endocardial & epicardial-specific *S1pr1* knockout	- Ventricular non-compaction - Ventricular septal defects Perinatal lethality	(55)
Cardiomyocyte-specific *S1pr2* knockout	- No alteration in coronary I/R injury	(56)
Constitutive mutant *s1pr2* allele (zebrafish, *mil*)	- Cardia bifida - Failure of cardiac progenitor migration to form primitive heart tube - Embryonic lethality	(22)
Cardiomyocyte-specific *S1pr3* knockout	- No alteration in coronary I/R injury	(57)
Constitutive *S1pr2* & *S1pr3* double knockout	- Increased infarct size after I/R injury - Perinatal lethality	(58, 59)
Constitutive *S1pr2* & *ApoE* double knockout	- Decreased atherosclerotic lesions - Decreased number of macrophages in lesions	(60)
Constitutive *S1pr3* & *ApoE* double knockout	- No change in atherosclerotic lesions - Decreased number of macrophages in lesions	(61, 62)
Constitutive *Cert* knockout	- Severely compromised cardiac function - Accumulation of ceramide - Embryonic lethality	(63)

## Sphingolipids in Cardiovascular Disease

Cardiovascular diseases (CVDs) are the leading cause of death in USA and worldwide, and it is estimated that by 2030 upward of 40% of the American population will be afflicted with some form of CVD (64). CVD occurs largely in the context of metabolic disease, such as diabetes, and obesity which are known to reconfigure sphingolipid profiles in multiple organs and tissues. It is unsurprising, then that a wide spectrum of sphingolipid species have been implicated in the pathophysiology of numerous CVDs (65, 66).

### Myocardial Lipotoxicity

It is now well-established that ceramide metabolism is altered in the context of type 2 diabetes mellitus (T2DM) and obesity which are both linked to CVD (67–69). In fact, higher plasma ceramide levels have been associated with visceral obesity, non-alcoholic fatty liver disease, and T2DM, which are also predictors of CVDs (70–76). Genetically modified mouse models of lipotoxicity have greatly facilitated understanding of sphingolipid contributions to lipotoxic cardiomyopathy (The outcomes of many of these lipotoxic animal models are summarized in Table 1). In fact, the first recognition of a potential link between sphingolipids and cardiac lipotoxicity arose from mice with cardiomyocyte-specific overexpression of long-chain acyl-CoA synthetase (77). This increased lipid uptake generated a lipotoxic cardiomyopathy phenotype. Hearts from these mice showed increased TAG concomitant with increased lipid droplets (77, 78). Because increased cell death was observed in these mice the investigators measured total ceramide, a known apoptotic mediator, and found a 50% increase in total ventricular ceramide content. Metabolic disease increases uptake, utilization, and storage of fatty acids in lipid droplets, but the potential toxicity of lipid droplets/TAG, or other lipids remained in question. To address this, a follow-up study crossed these transgenic mice with another strain overexpressing diacylglycerol acyltransferase 1 (DGAT1), which increased intracellular TAG and reduced ceramide (79). Because DGAT1 catalyzes the final step in TAG synthesis, it diverts Acyl-CoA into neutral sphingolipid pools, thereby reducing toxic lipids in myocardial lipotoxicity such as ceramides (80, 81). Long-chain acyl-CoA synthetase 1 (ACSL1) catalyzes the conversion of long-chain fatty acids to fatty acyl-CoAs, which are then used as substrates by SPT in *de novo* sphingolipid biosynthesis (82). Thus, crossing the DGAT1 mice with the acyl coenzyme A synthetase-1 (ACSL1) transgenic mice increased TAG and lipid droplets, but lowered ceramides and improved the cardiac phenotype of ACSL1 mice, indicating that triacylglycerols (TAGs) are not lipotoxic *per se* but serve as an indicator of lipid oversupply (83). These studies and others gave rise to the concept that routing of lipids into TAGs could decrease their incorporation into bioactive lipids and therefore improve cardiac outcomes of lipotoxicity; however, whether sphingolipids *per se* were the underlying toxic lipid species remained to be addressed.

The first studies to effectively identify a mechanistic link between ceramide and lipotoxic cardiomyopathy employed mice with transgenic overexpression of a GPI-anchored lipoprotein lipase on the cardiomyocyte surface (LpL^GPI^) (78). Lipoprotein lipase (LpL) degrades circulating TAGs into free fatty acids, thus increasing fatty acids to cardiomyocytes. Similar to the ACSL1 mice, these mice showed a robust lipotoxic cardiomyopathy phenotype. These mice were treated with an inhibitor of *de novo* sphingolipid biosynthesis, myriocin, which lowered ceramide and ameliorated the phenotype. Additionally, crossing the LpL^GPI^ mice to mice haploinsufficient in SPTLC1, which mediates *de novo* sphingolipid synthesis, showed a similar effect (16). These studies suggest that in the lipotoxic context, sphingolipid synthesis is deleterious, and increased incorporation of fatty acids into TAG is cardioprotective. This would imply that myocardial TAG levels are not indicative of cardiac dysfunction but rather reflect lipid metabolic dysfunction within the heart.

These studies were highly informative and led our lab to investigate specific sphingolipid species that may play roles in lipotoxic cardiomyopathy and the mechanism(s) by which they contributed. Based on our previous studies on the effects of saturated vs. unsaturated fatty acids on sphingolipid metabolism, we developed a high saturated fat diet (84–86). Mice on this high fat diet exhibited elevations in total myocardial ceramides and also DbCM-like cardiac hypertrophy and dysfunction (87). Further examination of specific ceramide chain lengths revealed that C14:0 ceramide as well as very long-chain (VLC) ceramides increased specifically in the high saturated fat-fed mice compared to mice on control and lard diets. Reducing sphingolipid synthesis by inhibition of SPT with Myriocin treatment inhibited sphingolipid synthesis including C14:0 ceramide production and also restored cardiac function. These animals also showed increased autophagosomes in cardiomyocytes; indeed, treatment of cultured primary cardiomyocytes with myristate (C14:0) increased autophagy. As noted above, ceramide synthase enzymes have partially distinct substrate preferences, and C14:0 ceramide is a product of CerS5, while VLC ceramides are products of CerS2. Indeed, overexpression of CerS5 in cardiomyocytes increased autophagic flux, and treating cardiomyocytes lacking CerS5 with myristate did not increase autophagy. These studies were the first to identify a specific ceramide species and ceramide synthase isoform in cardiac lipotoxicity. Upon overexpression of CerS2, VLC ceramides were elevated inducing insulin resistance, oxidative stress, mitochondrial dysfunction and mitophagy. As gain and loss-of function studies targeting CerS5 had no effect on these same functions, a distinct role was established for CerS2 (87, 88). These studies established specific roles for subsets of ceramide species and/or CerS enzymes in lipotoxic outcomes in the context of high fat feeding and subsequent diabetes (87, 88). Though these studies were conducted in mice and various primary and cultured cardiomyocyte models, the findings may nonetheless bear some relevance to humans (45). Importantly, many of these studies addressed lipotoxicity in the context of metabolic disease; however, other cardiac insults also cause lipotoxicity, and this may proceed by alternative mechanisms (69–76, 89). For example, metabolic tracing studies in mice subjected to pressure overload via transverse aortic constriction (TAC) showed that transgenic overexpression of acyl coenzyme A synthetase-1 (ACSL1) mitigated heart dysfunction relative to WT mice (83). In this context, ACSL1 overexpression prevented *de novo* synthesis of C16-, C24-, and C24:1-ceramides, which are synthesized by CerS5 and CerS2, respectively, but increased C20- and C22-ceramides. These subspecies can be generated by CerS4 and therefore, CerS4-derived ceramides may have a distinct, protective function in the context of HF, though this remains to be tested.

In addition to the n-acyl chain length, dictated by CerS, we showed that the sphingoid base chain length also distinguishes activities of sphingolipids. While the canonical SPT complex includes SPTLC1 and 2, SPTLC3 is an alternate subunit that can substitute for SPTLC2 and alter sphingoid base chain length. Therefore, the SPTLC1/2 complex generates an 18-carbon sphingoid base, but inclusion of SPTLC3 alters substrate preference to generate sphingoid bases of alternate chain lengths. We showed that high saturated fat feeding in mice induced SPTLC3 and altered cell sphingolipid profiles to include a high proportion of sphingolipids containing a 16-carbon sphingoid base within the myocardium (11). This shortened base did not cause autophagy but rather led to apoptotic cell death in cardiomyocytes, further exemplifying how distinct sphingolipid molecules can have divergent effects. Emerging literature continues to build a case for a role for SPTLC3 in human CVDs. Interestingly, a study of three German populations linked single nucleotide polymorphisms (SNPs) in the SPTLC3 locus to MI (90). Another study identified 28 SNPs close to the SPTLC3 locus which were significantly associated with reduced C22:0 and C24:0 ceramide concentrations, which are thought to correlate with disease risk (91).

Initial studies including our own showed that inhibition of overall sphingolipid biosynthesis prevented cardiac lipotoxicity, suggesting that merely reducing ceramide in the lipotoxic heart may be a “magic bullet.” However, rather than improving cardiac function, a cardiomyocyte-specific SPTLC2 null mouse showed an exacerbated cardiac phenotype (46). While the mechanism for this was not revealed in that study, a speculative hypothesis is that SPTLC3 may show compensatory upregulation in the context of SPTLC2 depletion. If so, this could explain the phenotype observed in the SPTLC2 depletion mouse model. These studies coupled with observations of cardioprotection in TAC correlating to increased C20:0 and C22:0 ceramides clearly demonstrate that roles of ceramides in cardiac pathology are complex. In addition to ceramides, however, alterations in dihydroceramides, ceramide-1-phopshates, sphingomyelins, and glycosphingolipids likely play disparate roles in cardiac pathology, and genetic manipulations or use of myriocin *in vivo* does not necessarily enable identification of specific lipid classes involved in pathological processes. Further research is required to fully understand the links between specific sphingolipid pools and molecular structures and deleterious outcomes through modulation of cell signaling.

### Coronary Artery Disease

Coronary Artery Disease (CAD) or IHD is the most common type of CVD worldwide and has been the leading cause of death for the past 16 years. CAD is caused by narrowing of arteries and subsequent reduction of blood flow to the heart due to build-up of plaque (atherosclerosis) within the arteries of the heart, ultimately leading to heart failure (HF). More often than not, coronary atherosclerosis observed in CAD occurs in the context of metabolic disease. As a result of chronic CAD, myocardial infarction (MI) and HF often occur. In contrast to much literature implicating ceramides as inducers of CVDs, pronounced cardiogenic and cardioprotective properties have been attributed to S1P (92–101).

Current therapeutics already undergoing clinical trials for CAD, ischemia/reperfusion injury, MI and HF target the S1P/SphK axis, specifically drugs targeting S1P receptors (102–104). Mice with combined deletion of S1PR2 and S1PR3 subject to ischemia/reperfusion injury showed increased infarct size, however, infarct size was not altered when either S1PR2 or S1PR3 was deleted (58, 105). However, another study showed that intravenous SPC treatment of S1PR3 null mice subject to IR injury reduced infarct size (106). Agonists specific to S1PR1 protected mouse cardiomyocytes from hypoxia, while the opposite effect was observed with S1PR1 antagonists (107–111). Another study showed that S1PR1 attenuated cardiac fibrosis and hypertrophy in mice with HF induced by TAC (112). Nogo-A/B deficient mice were protected from HF for up to 3 months after TAC, while also seeing a significant induction of S1P (14).

In mouse, rabbit and rat models of ischemia/reperfusion injury (IRI) it was noted that ceramide, membrane neutral sphingomyelinase (nSMase) and acidic sphingomyelinase (aSMase) increased in the infarct site and blood with concomitant decrease of S1P (113–120). Ischemic preconditioning with S1P is a tried and proven method to induce significant recovery of cardiac function and infarct reduction in IRI (57, 121–123). The absence of improved cardiac function in SphK1 and SphK2 ablated mice subject to IR levels suggest the significance of S1P in ischemic conditioning (121, 124). These studies suggest targeting the S1P-SphK axis satisfies the criterion as an effective therapeutic agent to overcome the damages elicited by IRI. Though further investigation into the crosstalk between S1P receptors and analogs signaling behavior in comorbidities would better optimize the therapeutic potential of S1P in IRI.

Multiple *in vitro* studies observed upregulation of aSMase and nSMase along with increased SM in animal models of HF (125, 126). The nSMase and aSMase hydrolyze sphingomyelin to release ceramide, and thus the accumulation of ceramide in post-ischemic heart may arise from SM catabolism and not *de novo* sphingolipid biosynthesis (127, 128). Another study in both mice and humans with HF noted increased levels of SPTLC2, which participates in *de novo* sphingolipid synthesis and likely contributes to the significant increase of total ceramides in the aforementioned studies (45). However, this same study did not note any changes in aSMase or nSMase in HF, the primary catabolic enzymes for ceramide production (45). Therefore, it may be that chronic conditions leading to HF increase *de novo* synthesis, while acute insults such as MI activate sphingolipid catabolism, though there are clear exceptions to this concept, including nSMase activity was increased 2–3 months post-MI and SPTLC2 was observed to increase 2 weeks post-MI (45, 127).

While the roles of ceramides and S1P are well-established and antagonistic in CVDs, some literature suggests sphingosine plays a dichotomous role as a cardio protectant and CVD inducer. Induction of sphingosine in patients with and animal models of IRI points toward the maladaptive role of sphingosine in CVDs (129, 130). However, the same group preconditioned animals with sphingosine prior to ischemia or perfusion resulting in massive reduction of infarct size. This contradictory evidence points toward sphingosine as a cardio protectant in CVDs (131). Importantly, sphingosine can be used as a substrate for generation of either ceramides or S1P; therefore, whether it is protective or deleterious may arise from its metabolic fate. However, sphingosine does have its own signaling functions and thus may make these contributions directly and without further metabolism (132–134).

## Sphingolipids as Emerging Biomarkers in Assessing Cardiovascular Disease

Over the past several decades various heart studies including the Framingham Heart Study, Busselton Family Heart Study, Strong Heart Family Study, Utah CAD and others have sought to determine CVD development, risks and therapeutics all aimed at combating CVD. As a result, conventional risk factors such as age, sex, ethnicity, blood pressure, total triglyceride levels and total cholesterol levels emerged as biomarkers for risk of major adverse cardiac events. However, given the substantial and rising burden of CVDs, ongoing efforts to shed light on novel, more specific biomarkers for CVD are needed. In response to this need, more sensitive lipidomics analysis have been developed focusing on sphingoid base and acyl chain length composition of total sphingolipids. Using these advanced techniques, risk assessment scores utilizing sphingolipid species levels have been recently developed in detecting CVDs and they continuously outperform conventional cardiovascular risk markers (135). For example, A Busselton Family Heart Study identified many classes of the sphingolipid species ceramides, DHC, mono-, di- and tri-hexosylceramides, SM, GM1, GM3 and sulfatides associated with heritable cardiac events, most of which were positively genetically correlated with LDL, HDL, total cholesterol and negatively correlated with triglyceride levels. In another study, the serum of patients with a clinical diagnosis of HF with preserved ejection fraction (HFpEF), the most common form of HF and also strongly associated with diabetes, in The Alberta Heart Failure Etiology and Analysis Research Team (HEART) project showed reduced SM compared to non-HF controls (136). These sphingolipids seemed to have no relationship to conventional risk factors such as diastolic blood pressure, systolic blood pressure, BMI and waist-hip ratio, suggesting they may be used as more specific markers to identify high-risk patients especially likely to have CVD (137–140).

A growing body of literature suggests long-chain ceramides and very long-chain ceramides and SM are associated with adverse cardiac outcomes. Ceramide analysis on an aggregate of 29,818 individuals from 7 cohort studies and determined plasma Ceramide (d18:1/16:0), (d18:1/18:0), and (d18:1/24:1) levels were associated with major adverse CVD events. Whereas, Ceramide (d18:1/22:0) were not associated (141). Javaheri et al., observed significant association of increased circulating concentrations of C16:0 and C18:0 ceramides in participants with HFpEF (142). This study was especially informative, as HFpEF is difficult to diagnose and controversy still exists as to diagnostic algorithms (143). Similarly, another study showed that levels of ceramide and SM with 16-carbon acyl chain length were directly associated with higher risk of mortality deaths from CVD (144). Analysis of myocardial tissue and serum from patients with HF showed increased ceramide, the significance of which was mainly driven by the very long chain ceramides (45). When the HFpEF patients underwent left ventricular assist devices (LVAD) these changes were reversible. In contrast, another study observed that levels of Ceramide with a 22-, or 24-carbon acyl chain length and SM with a 20-, 22-, or 24- carbon acyl chain were directly associated with lower risks of CVD mortality (142, 144).

While these biomarkers and diagnostic indicators represent advances in identifying and diagnosing CVD, the distinction between long chain (i.e., C16–C18) and very long chain (i.e., C20–C24) ceramides, which are generated by distinct ceramide synthase isoforms (CerS1/5/6 vs. CerS2/4, respectively) hints at potential mechanistic involvement. However, though much circulating lipid in general arises from liver, a detailed study demonstrated that circulating ceramides but not SM are derived from the endothelium of blood vessels (145). Interestingly the endothelium is functionally impaired in CVDs and could thus contribute to the plasma sphingolipid profile reported in HF. Therefore, further determining the points of origin of these lipids may enable further study on potential mechanistic functions of these lipids in CVD.

## Discussion

In this review we highlighted recent studies implicating sphingolipids in heart development, ischemic injury, and CVDs. In general, the SphK/S1P/S1P receptor signaling axis appears beneficial both in development and in ischemic injury. On the other hand, elevation of myocardial ceramides appears mostly deleterious. Therapeutic potentials of targeting the SphK/S1P/S1P and ceramide pathways are beneficial and have been approved for treatment in patients in other systems, for example the FDA approved FTY-720, an S1P antagonist for treating relapsing multiple sclerosis (146). Since these have been tried in other systems they can be applied to CVDs, as targeting this axis in animal models of CVDs suggested prevention or reversal (147). Targeting CerS1-6 have a great therapeutic potential but there have been multiple developmental roadblocks, however a recent study observed that a CerS1 selective inhibitor endogenously inhibited mitochondrial fatty acid oxidation in muscle and regulated whole-body adiposity, which could potentially benefit in treating patients with some forms of CVDs (148). Further, relationships among circulating sphingolipid species are novel biomarkers of CVD. Therefore, sphingolipid metabolism and signaling is a constant thread from heart development to CVD, with distinct pathways playing beneficial or deleterious roles.

Lipidomic analyses based on mass spectrometry has enabled identification of different sphingoid bases and acyl chain lengths allowing for novel biomarkers in diagnosing and assessing risk in the context of CVDs. These novel risk scores and biomarkers that utilize sphingolipids show the full complexity of the altered lipid metabolism and outperform traditional lipid measurements. Sphingolipid risk scores are better predictors of major adverse cardiac events than conventional risk factors, including total and LDL cholesterol. As such, ceramide testing is now routinely performed as a diagnostic tool in CVD (149–152).

Many studies highlighted in this review contradict one another with respect to the significant sphingolipid species associated with a particular heart development stage or in association with a CVD. It is important to note these controversial observations can arise from the type of lipidomics technology utilized, the size of the population studied, the type of sample collected, sex distribution, and race/ethnicity. For example, an African American healthy control population had significantly higher plasma levels of most SM species and lower levels of lactosylceramide species compared to Caucasian control subjects in the same study (153). These differences were noted in healthy individuals; therefore, stratification of a study population based on race/ethnicity is essential to provide clear conclusions and, moreover, to identify health disparities when considering disease patients between different ethnicities.

Implications of the association of SPTLC3 SNPs with MI and other cardiovascular events is an emergent finding deserving further mechanistic study (90, 91). Synthesis of d16-, and d20-sphingoid based sphingolipids are entirely dependent while d18-sphingoid based sphingolipids are partially dependent on SPTLC3. Our lab previously showed that 1/3^rd^ of the mouse myocardial sphingolipid pool is comprised of d16-sphingoid based sphingolipids presumably derived from SPTLC3 (11). Moreover, HF patients showed reduced SPTLC3 upon placement of left-ventricular assist devices (LVAD) (45). In addition to recognizing the function of SPTLC3 to generate non-canonical sphingoid bases, our understanding of the complexity of sphingolipid metabolism and the diversity of sphingolipid species has exploded in recent years. Thus, when it comes to matters of the heart, untargeted sphingolipidomics has the unique potential for revealing non-canonical sphingolipid species that indicate and/or play mechanistic roles in CVD.

Importantly, while most *in vivo* studies in the heart that address sphingolipid function have manipulated SPT, either genetically or pharmacologically (e.g., with myriocin treatment), these approaches inhibit biosynthesis of all sphingolipids, both desirable and deleterious, and, therefore, would be much too broad for clinical application. However, identification of specific enzymes that participate in distinct branches and pathways of sphingolipid metabolism would provide much greater specificity for therapeutic intervention in the CVD context. However, the dearth of isoform-specific CerS inhibitors has been an impediment to therapeutic targeting of CerS, though efforts to develop specific agents are beginning to yield results (148).

Over the last decade our lab has focused on studying heart sphingolipids showing that ceramides with not only distinct N-acyl-chains but particular sphingoid base backbone lead to apoptosis, mitochondrial damage and lipotoxicity in cardiomyocytes. It should be noted that identification of specific CerS isoforms that mediate CVD does not necessarily implicate ceramides, *per se*, but could implicate dihydroceramides as well as downstream products of ceramides including ceramide-1-phosphate, O-acylceramides, sphingomyelin, and glycosphingolipids. This understanding is crucial both for enabling further specificity of therapeutic targeting and facilitating the potential identification of additional therapeutic targets. Ultimately, more research is needed to elucidate the regulatory pathways by which sphingolipids regulate cardiogenesis and cardiovascular function in both health and disease.

## Author Contributions

LC and AK: writing—review and editing, visualization, and conceptualization. LC, AK, and MJ: writing—original draft and data curation. LC: supervision and project administration. All authors contributed to the article and approved the submitted version.

## Conflict of Interest

The authors declare that the research was conducted in the absence of any commercial or financial relationships that could be construed as a potential conflict of interest.
